# A socio-interpersonal approach to adjustment disorder: the example of involuntary job loss

**DOI:** 10.1080/20008198.2018.1425576

**Published:** 2018-01-31

**Authors:** Louisa Lorenz, Axel Perkonigg, Andreas Maercker

**Affiliations:** ^a^ Department of Psychology, University of Zurich, Zurich, Switzerland

**Keywords:** Adjustment disorder, socio-interpersonal model, job loss, ICD-11, stress-response syndrome, path analysis, trastorno de adaptación, Modelo Socio-Interpersonal, Perdida de trabajo, CIE-11, Síndrome de respuesta frente al estrés, Análisis de ruta, 适应障碍, 社会人际模型, 失业, ICD-11, 压力反应综合征, 路径分析, • The reconceptualization of adjustment disorder as stress-response syndrome for ICD-11 requires new research into its aetiology.• The socio-interpersonal framework model emphasizes the social reality of the individual affected by a stressful life event.• Individuals affected by involuntary job loss frequently report adjustment disorder symptoms, 25.6% met the diagnostic criteria.• Loneliness, dysfunctional disclosure, and self-efficacy were associated with adjustment disorder symptom severity and diagnostic status.• The consideration of the social context of an individual affected by involuntary job loss may improve service provision.

## Abstract

**Background**: Adjustment disorder (AjD) was redefined for ICD-11 with core symptoms of preoccupation with a stressor and failure to adapt. The socio-interpersonal framework model for stress-response syndromes suggests that interpersonal factors, besides intrapersonal processes, substantially contribute to the development of AjD.

**Objective**: The current study aimed to identify predictive factors in the development of AjD symptoms by the application of a framework model for stress-response syndromes.

**Method**: *N* = 321 recently laid-off participants (47.7% female) were assessed with a newly developed standardized clinical diagnostic interview section on ICD-11 AjD. Self-report questionnaires measured AjD symptom severity, and interpersonal and intrapersonal predictors. Path analysis was used to model the associations between AjD symptom severity and the predictor variables. We conducted logistic regression to identify associated characteristics of diagnostic status.

**Results**: AjD symptoms were highly prevalent and 25.6% of participants met the diagnostic criteria. Higher loneliness, higher dysfunctional disclosure, and lower self-efficacy were associated with both higher symptom severity and higher likelihood of meeting the diagnostic criteria for AjD. Higher perceived social support was associated with higher likelihood for AjD diagnosis.

**Conclusions**: Research on risk factors for AjD is still sparse. This study provided empirical evidence on the role of interpersonal factors supporting the socio-interpersonal model for stress-response syndromes.

## Abstract (es)

**Planteamiento**: El trastorno de adaptación (TAd) se redefinió para la CIE-11 con síntomas centrales de preocupación por un factor estresante y falta de adaptación. El modelo de marco socio-interpersonal para los síndromes de respuesta ante el estrés sugiere que los factores interpersonales, además de los procesos intrapersonales, contribuyen sustancialmente al desarrollo del TAd.

**Objetivo**: El presente estudio pretendía identificar los factores predictivos en el desarrollo de síntomas de TAd mediante la aplicación de un modelo de marco de trabajo para los síndromes de respuesta frente al estrés.

**Método**: Se evaluaron N = 321 participantes recientemente despedidos (47,7% mujeres) con una sección de entrevista diagnóstica clínica estandarizada recientemente desarrollada para el TAd de la CIE-11. Los cuestionarios de autoinforme midieron la gravedad de los síntomas de TAd y los predictores interpersonales e intrapersonales. El análisis de ruta se utilizó para modelar las asociaciones entre la gravedad del síntoma de TAd y las variables predictoras. Realizamos una regresión logística para identificar las características asociadas del estatus del diagnóstico.

**Resultados**: Los síntomas de TAd fueron altamente prevalentes y el 25.6% de los participantes cumplieron con los criterios diagnósticos. Una mayor soledad, una revelación personal más disfuncional y una menor autoeficacia se asociaron con una mayor gravedad de los síntomas y una mayor probabilidad de cumplir los criterios de diagnóstico para el TAd. Un mayor apoyo social percibido se asoció con una mayor probabilidad de diagnóstico de Tad.

**Conclusión**: La investigación sobre los factores de riesgo para el TAd aún es escasa. Este estudio proporcionó evidencia empírica sobre el papel de los factores interpersonales que apoyan el modelo socio-interpersonal para los síndromes de respuesta frente al estrés.

## Abstract (zh)

**背景：**适应障碍（AjD）在 ICD-11被重新定义，其核心症状为‘面对应激源而无法适应’。压力反应综合征的社会人际框架模型提出，人际因素和自我过程一起显著影响了AjD的发展。

**目标：**本研究旨在通过应用一个压力反应综合征的框架模型识别AjD症状发展的预测因素。

**方法：**使用一个新开发的标准化临床诊断访谈评估321名最近被解雇的被试（47.7%为女性）的 ICD-11 AjD。自评问卷测量AjD症状的严重度，人际间和自我相关的预测指标。使用路径分析建立AjD症状严重度和预测指标间的关系模型。使用 logistic 回归来识别诊断状态相关的特征变量。

**结果：**AjD症状高发，25.6%的被试符合诊断标准。更高孤独感和非功能性暴露、更低的自我效能感和高症状严重度相关，也更可能达到AjD诊断标准。更高的知觉到的社会支持和达到AjD诊断标准的更高可能性相关。

**结论：**关于AjD的风险因素的研究还很稀少。本研究提供了关于人际因素的实证证据，支持了压力反应综合征的社会人际模型。

During the latest revision of the International Classification of Diseases (ICD) and the Diagnostic and Statistical Manual of Mental Disorders (DSM), the adjustment disorder (AjD) diagnosis has been reconceptualized as a stress-response syndrome (Maercker et al., 2013; Strain & Friedman, 2014). Stress-response syndromes are defined as an exaggeration of a stress response that can lead to mental illness (Horowitz, 1986). AjD describes the development of emotional or behavioural symptoms in response to a critical life event or external life stressor of minor intensity. It can occur after non-traumatic but serious acute or chronic life events such as an involuntary job loss (World Health Organization, 1992). The upcoming ICD-11 by the World Health Organization (WHO) proposes two core symptoms consisting of preoccupation and failure to adapt (Maercker et al., 2013). Preoccupation with the stressor is described as a state of recurring distressing thoughts about the event or its consequences, and of constant rumination. Failure to adapt symptoms subsume generalized stress-response symptoms, such as sleep disturbances or concentration problems (Maercker et al., 2013). Accessory symptoms, such as avoidance, anxiety, depressive symptoms, or impulsivity, can occur (Maercker et al., 2013).

Recent studies provided evidence for the proposed stress-response conceptualization for ICD-11 regarding its reliability and clinical utility (Bachem, Perkonigg, Stein, & Maercker, 2016; Glaesmer, Romppel, Brähler, Hinz, & Maercker, 2015; Keeley et al., 2016; Zelviene, Kazlauskas, Eimontas, & Maercker, 2017). However, little is known about predictive factors or models for the development of this disorder. Studies found that female gender was a risk factor for adjustment problems in cancer patients (e.g. Hund et al., 2016). Further studies identified younger age, worse preceding mental health, higher alexithymia, neuroticism, psychoticism, harm avoidance, and lower self-transcendence as predictors of AjD in military recruits (Chen, Chen, Chen, & Lung, 2011; Lung, Lee, & Shu, 2006; Na et al., 2012). Focusing on neurobiology, studies found decreased grey matter volume (Myung et al., 2016) and an increased sensitivity of the bimodal P300 amplitude (Kajosch et al., 2016) in patients diagnosed with AjD. Furthermore, various studies found interpersonal variables predicting AjD, e.g. lower cooperativeness (Chen et al., 2011), higher interpersonal distance, higher social diversion, and lower social support (Ponizovsky, Levov, Schultz, & Radomislensky, 2011). However, few studies investigated psychological ‘intrapersonal’ factors, such as general self-efficacy (Fankhauser et al., 2010), self-esteem (Ponizovsky et al., 2011), and cognitive reappraisal (Hu et al., 2014) in the context of stress-response.

## 1. Socio-interpersonal model of stress-response syndromes

Interpersonal relationships play an important role in regulating individual well-being (Antonucci, Ajrouch, & Birditt, 2014). They can differ in their closeness, quality, and structure, and may have different impacts on mental health (Antonucci et al., 2014), especially after stressful life events (Cohen & Wills, 1985). As indicated, initial empirical evidence exists that interpersonal factors are of particular importance for developing and maintaining AjD. The socio-interpersonal framework model by Maercker and Horn (2013) was developed for stress-response syndromes. It assumes that individuals are nested in different levels of social contexts that influence the recovery after extreme stress experiences. The first level includes social affective and related intrapersonal processes. Social affective reactions are affective states that refer to both self and others (e.g. Orth, Robins, & Soto, 2010), such as shame, anger, guilt, and loneliness (Hawkley & Cacioppo, 2010; Maercker & Horn, 2013). The second level of the socio-interpersonal framework model captures interaction processes in close relationships, such as social support, empathy, and communication factors. Higher perceived social support was shown to be predictive of better mental health among crisis aid workers (Prati & Pietrantoni, 2010), and of less adjustment problems in cancer patients (Rizalar, Ozbas, Akyolcu, & Gungor, 2014). Likewise, the quality of social support resources seems to influence the psychological adjustment outcome after stress exposure (Ajrouch, Abdulrahim, & Antonucci, 2013; Brewin, Andrews, & Valentine, 2000). Disclosure of stressful experiences has been shown to facilitate recovery from severe stress (Freedman, Gilad, Ankri, Rozier, & Shalev, 2015; Pennebaker, 1989; Pielmaier & Maercker, 2011). The third level includes societal and cultural factors. Müller, Forstmeier, Wagner, and Maercker (2011) found that societal value orientations were directly and indirectly predictive of grief reactions and adjustment disorder symptoms.

There is some evidence for the validity of the socio-interpersonal framework model in different contexts of stress-response. Higher dysfunctional disclosure, lower social acknowledgement, and higher co-rumination significantly predicted secondary PTSD symptoms in Belarusian rescue workers (Krutolewitsch, Horn, & Maercker, 2016). Maercker, Hilpert, and Burri (2016) found in former indentured child laborers that higher dysfunctional disclosure was associated with less life satisfaction, higher perceived social support was associated with less depressive symptoms, and higher social acknowledgement was associated with an increase in depressive symptoms over time. Furthermore, the risk of an AjD after a stressful life event for men was elevated when their female partner showed clinically significant symptoms of depression, and higher depressive symptoms in the female partner were associated with higher preoccupation in the male partner (Horn & Maercker, 2015). Fankhauser et al. (2010) found that motivation regulation and general self-efficacy mediated the negative relationship between social acknowledgement and AjD symptom severity, and that the reluctance to talk mediated the negative relationship between general self-efficacy and AjD symptom severity. These results support the view that contextual factors should be incorporated in research on stress-responses.

In the past, the AjD diagnosis had only been defined via the exclusion of other mental disorders, which resulted in too little research on the diagnostic features, its aetiology, and treatment (e.g. Baumeister & Kufner, 2009). Since AjD has been reconceptualized as a stress-response syndrome, the socio-interpersonal framework model should be applicable to this disorder. This creates the opportunity to investigate etiological factors in the development of the disorder based on theoretical assumptions. A prerequisite for a comprehensive analysis of interpersonal and intrapersonal predictors of AjD as proposed for ICD-11 would be a large enough sample with a homogeneous stressor event. Job loss is one of those critical life events that is frequent and can be regarded as example constellation for AjD. Research has shown its negative impact on physical health (Gallo et al., 2004), health behaviour (Gallo, Bradley, Siegel, & Kasl, 2001), and mental health (Ziersch, Baum, Woodman, Newman, & Jolley, 2014), in particular the onset of depressive symptoms and anxiety reactions (Barbaglia, Have, Dorsselaer, Alonso, & De Graaf, 2014).

The current study intends to contribute empirical evidence for AjD as redefined for ICD-11. The first aim was to identify predictive factors for AjD symptom severity based on assumptions of the socio-interpersonal framework model and previous empirical evidence. It was expected that emotion regulation and self-efficacy as intrapersonal processes, and social support, loneliness, and dysfunctional disclosure as interpersonal processes, would be associated with AjD symptom severity. The second aim of the present study was to investigate the association of the same intra- and interpersonal characteristics with AjD diagnostic status.

## 2. Method

### 2.1. Participants and procedure

The data for this analysis derived from the Zurich Adjustment Disorder Study, a longitudinal study cross validating the proposed AjD diagnosis for ICD-11 and DSM-5. The study was approved by the Ethics Committee of the University of Zurich in June 2015. Recruitment of participants took place from September 2015 to August 2016 in the greater Zurich area. Most of the participants were recruited via regional job centres. The personnel consultants handed out the study information or an advertising flyer to individuals eligible for participation. Interested individuals could then contact the study coordinator for further information and enrolment in the study. Other means of recruitment were three local newspaper articles and a mailing list of the University of Zurich for people generally interested in study participation. All participants have been laid off within nine months prior to participation. People were excluded from the study if they did not speak German fluently, were aged under 18 years, were unable to give written informed consent, or suffered from a severe mental illness. Interviews were conducted either at the University or at participants’ home. After being informed about the aims and the procedure of the study, participants gave their written consent. A total of 463 people showed interest in study participation. Ninety-eight of them did not meet the inclusion criteria, 21 potential participants could not be reached again, and 10 people did not agree to participate for other reasons. This led to a total sample of 334 participants included in the study. The demographic characteristics of the sample can be found in Table 1. Gender was equally distributed across the sample (52.3% male; 47.7% female). The male sample was slightly older than the female sample (*t*(319) = 2.08, *p *= .039).

**Table 1.** Demographic characteristics of participants (*N* = 321).

	Full sample		Male		Female	
	*M*	*SD*	*M*	*SD*	*M*	*SD*
Age, years	43.70	10.64	44.88	10.44	42.42	10.76
Time since job loss, months	3.31	1.96	3.36	1.99	3.26	1.92
	Full sample		Male		Female	
	*n*	%	*n*	%	*n*	%
Job status						
Started a new job	26	7.8	10	6.0	15	9.8
Still unemployed	292	91.0	156	92.9	136	88.9
No information	4	1.2	2	1.2	2	1.3
AjD prevalence	81	25.6	35	21.1	46	30.7

AjD Prevalence is based on *n* = 316 participants due to missing data.

### 2.2. Measures

The *Diagnostic Status of AjD* was assessed by a modified version of the computer-assisted Munich Composite International Diagnostic Interview (M-CIDI; Wittchen & Pfister, 1997). The M-CIDI is a valid and reliable standardized clinical interview for the assessment of symptoms, syndromes, and diagnoses according to DSM-IV and ICD-10 (Wittchen, Lachner, Wunderlich, & Pfister, 1998; Wittchen & Pfister, 1997). To determine the diagnostic status of AjD, a new AjD CIDI-module was designed (Perkonigg, Strehle, Lorenz, Beesdo-Baum, & Maercker, 2015). In a first step, it assesses all events occurring within 12 months prior to the interview (including event characteristics). Next, the module asks for ICD-11 and DSM-5 symptoms occurring in response to the most severe event as indicated by the participant. In a third step, it assesses onset and recency of symptoms, and impairment due to the symptoms.

*AjD Symptom Severity* was assessed using the Adjustment Disorder – New Module 20 (ADNM-20; Einsle, Köllner, Dannemann, & Maercker, 2010). The ADNM-20 is a self-report questionnaire that evaluates previous life events and AjD symptoms in response to the most severe life event (Einsle et al., 2010). In the present study, we used a contextualized version of the ADNM-20 symptom list and all the items referred to the job loss. The response format is a 4-point Likert scale ranging from 1, *never*, to 4, *often*. We used the eight items that measure preoccupation, failure to adapt, and functional impairment to build a total sum score (ADNM-8). The ADNM-20 showed satisfactory properties regarding factor structure, internal consistency, retest-reliability, and construct validity (Bley, Einsle, Maercker, Weidner, & Joraschky, 2008; Einsle et al., 2010; Glaesmer et al., 2015) in previous studies. The use of the ADNM-8 found initial support in two previous studies (Kazlauskas, Gegieckaite, Maercker, Eimontas, & Zelviene, 2018; Zelviene et al., 2017). The internal consistency in this study was α = .87.

*General Self-Efficacy* was measured using the General Self-Efficacy Scale (GSE; Schwarzer & Jerusalem, 1999). It consists of 10 items that are answered on a 4-point Likert scale ranging from 1, *not correct*, to 4, *absolutely correct*. The total score is calculated by using the sum of all variables. The GSE showed high internal consistencies of α = .75–.91 and satisfactory discriminant and convergent validity (Hinz, Schumacher, Albani, Schmid, & Brähler, 2006; Schwarzer & Jerusalem, 1999). The internal consistency in this study was α = 89.

*Emotion Regulation Competencies* were assessed with the Emotion Regulation Questionnaire (ERQ; Gross & John, 2003). Ten items assess reappraisal and suppression on a 7-point Likert scale ranging from 1, *don’t agree*, to 7, *agree absolutely*. The items are aggregated on two subscales using the mean of the respective items. The English version showed internal consistencies between α = .68–.82 in different studies and the retest-reliability was r_tt_ = .69 over a period of three months (Gross & John, 2003). The German translation showed comparable psychometric properties (Abler & Kessler, 2009). The internal consistency in this study was α = .66 for reappraisal and α = .87 for suppression.

*Loneliness* was measured using a composite score of two single items from other scales. The first item of the loneliness scale derived from the Brief Symptom Inventory–18 (BSI-18; Spitzer et al., 2011). The item formulation was *‘How strong did you experience feelings of loneliness during the past 7 days?’* and it was answered on a 5-point Likert scale ranging from 0, *not at all*, to 4, *very strong*. The second item derived from the Social Functioning Questionnaire (SFQ; Tyrer et al., 2005). The item formulation was *‘I feel lonely and isolated from other people’* and it was answered on a 4-point Likert scale ranging from 0, *almost all the time*, to 3, *not at all*. This item was recoded before building the sum score with the other item of the scale. The internal consistency of this short loneliness scale was α = .75.

*Dysfunctional Disclosure* was measured using the Disclosure Questionnaire (Mueller & Maercker, 2006) in an abbreviated form (Pielmaier & Maercker, 2011). The 12 items can be divided into three subscales: *urge to talk, reluctance to talk*, and *emotional reactions while disclosing*. The response format is a 6-point Likert scale ranging from 0, *not at all*, to 5, *absolutely*. The total score is formed by summing up the individual items either to the subscales or the whole scale. In the long version, Cronbach’s α ranged between .82–.87 and the retest-reliability in a period of 1–3 months ranged between r_tt_ = .76–.89 for the subscales (Mueller, Beauducel, Raschka, & Maercker, 2000). The internal consistency in this study was α = .79.

*Perceived Social Support* was assessed using the Social Support Questionnaire, short form – German (FSozU-K; Fydrich, Sommer, Tydecks, & Brähler, 2009). It consists of 14 items that are answered on a 5-point Likert scale ranging from 1, *don’t agree*, to 5, *agree*. The total score is built by the mean of all items answered to avoid problems with missing data (Fydrich et al., 2009). The FSozU-K showed high internal consistency (α = .94), a high retest reliability over a period of one week (r_tt_ = .96), and satisfactory discriminant and convergent validity (Fydrich et al., 2009). The internal consistency in the present study was α = .90.

*Positive and negative support resources* were assessed with items from the Daily Hassles Scale (Perkonigg & Wittchen, 1995). Six items each measured positive and negative support from partner, children, parents, siblings, friends, and neighbours. The response format was a 4-point Likert scale ranging from 1, *often*, to 4, *never*. In order to facilitate interpretation, all items were reverse coded, so that a higher score indicated a more positive or more negative support resource. Total scores were computed using the mean of all items. The internal consistency was α = .65 and α = .68 for positive and negative social support, respectively.

### 2.3. Data analysis

Statistical analyses were conducted using IBM SPSS Statistics, Version 23, and MPlus, Version 8 (Muthén & Muthén, 1998–2017). We performed multivariate outlier analysis using the Mahalanobis distance (Penny, 1996). Five cases were excluded from the analysis because they were multiple outliers on the scales of interest. Furthermore, six cases were excluded from the analysis because they showed a *z*-score > 3.29 on at least one of the scales (Field, 2013). The final sample size for the analysis was *N =* 321. Four cases did not have data of the CIDI due to technical problems with the computer program. One participant refused to answer any questions in the AjD module. Hence, the sample size for the logistic regression was reduced to *n =* 316.

#### 2.3.1. Path model

**Figure 1.**
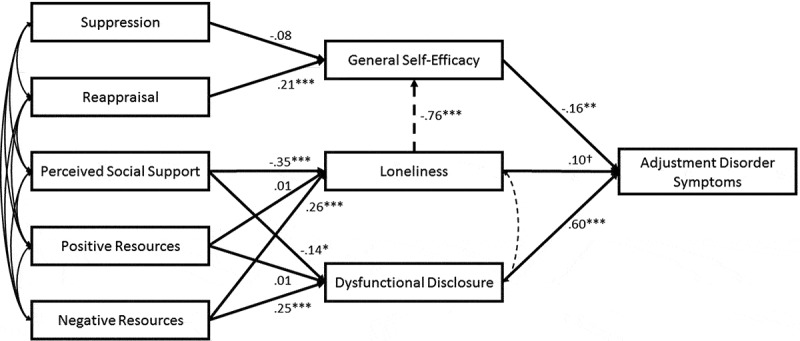
Final path model predicting adjustment disorder symptomatology. *N* = 321. The figure displays standardized path coefficients between intra- and interpersonal predictors and adjustment disorder symptoms. Broken lines indicate changes between the initial and the final model based on modification indices. Double headed arrows between endogenous variables indicate correlations between residual variances. All correlations were significant, except for reappraisal and negative resources. ^†^ *p* < .06; *p < .05; **p < .01; ***p < .001.

To investigate the relationship between predictor variables and AjD symptom severity as outcome, we conducted a path analysis. We formulated an initial model with general self-efficacy (intrapersonal), loneliness, and dysfunctional disclosure (both interpersonal) as proximal predictors of AjD symptom severity. These variables refer to the first level of the socio-interpersonal model and were thus expected to be directly linked to AjD symptom severity. We further included suppression and reappraisal (both intrapersonal) as emotion regulation strategies. The second level of the socio-interpersonal framework model was represented by perceived social support, and positive and negative support resources (all interpersonal). They served as distant, exogenous variables in the model. In a first step, we formulated a restricted model (Figure 1, unbroken lines), in which the effects of the intra- and interpersonal variables were separated. Based on the modification indices, we allowed further predictions and covariations between the predictors in subsequent steps (Figure 1, broken lines). We used the robust maximum likelihood (MLR) estimator for model estimation. Standard recommendations for assessing model fit of the final model were followed (Hu & Bentler, 1999): a chi-square to degree of freedom ratio (χ^2^:df) of less than 3:1, a Comparative Fit Index (CFI) > .90, a Tucker-Lewis Index (TLI) > .90, and a Root-Mean Square Error of Approximation with 90% confidence intervals (RMSEA) and Standardized Root Mean Square Residual (SRMR) < .08 were defined as acceptable. The Bayesian Information Criterion (BIC) was used to compare relative model fit and the model with the lowest BIC was considered best fitting.

#### 2.3.2. Logistic Regression

Logistic regression analysis was used to investigate predictors of AjD diagnostic status. In scales that used sum scores, missing value imputation was performed using the mean of the remaining items on that scale for the respective person (Little & Rubin, 2002). No missing values were imputed in scales that used mean scores. We calculated a model containing gender, age, and the same predictor variables as in the path model. The resulting B values of the logistic regression were transformed into standardized β weights (King, 2007).

## 3. Results

### 3.1. Descriptives

The prevalence of AjD was 25.6% (*n *= 81), with a marginally significant higher proportion of women (30.7%) being diagnosed than men (21.1%; χ^2^(1) = 3.80, *p *= .051). Women on average also showed higher AjD symptom severity than men (*t*(309) = −2.60, *p *< .05). For 23.1% (*n =* 73) the job loss was the only event they reported, 30.4% (*n =* 69) reported having experienced one life event besides the job loss within the past year, 21.5% (*n =* 68) reported two other life events, and 25.0% (*n =* 35) experienced three or more other life events in the 12 months before the interview. The most prevalent life events besides the job loss were illness or death of a loved one (35.5%, *n =* 112), financial problems (31.6%, *n =* 100), and family conflicts (28.5%, *n =* 90). The correlation coefficients between the study variables can be found in Table 2.

### 3.2. Path model

Figure 1 provides the path model for the prediction of AjD symptom severity irrespective of diagnostic status. The initially specified restricted model exhibited insufficient model fit across all indices (*Model 1*: χ^2^ = 113.8, *df* = 15; CFI = .76, TLI = .58, RMSEA 90% CI = .14 [.12;.17], SRMR = .10; BIC = 6801.7). The first modification included the regression of self-efficacy on loneliness and model fit was improved (*Model 2*: χ^2^ = 67.9, *df* = 14; CFI = .87, TLI = .76, RMSEA = .11 [.09;.14], SRMR = .07; BIC = 6758.8). As a second modification, the correlation between the residual covariances of loneliness and dysfunctional disclosure was freely estimated and model fit was again improved (*Model 3*: χ^2^ = 39.5, *df* = 13; CFI = .94, TLI = .87, RMSEA = .08 [.05;.11], SRMR = .04; BIC = 6735.6). The third modification allowed the correlation between the residual covariances of loneliness and self-efficacy to be freely estimated and fit of the final model was excellent across all indices (*Model 4*: χ^2^ = 16.0, *df* = 12; CFI = .99, TLI = .98, RMSEA = .03 [.00;.07], SRMR = .03; BIC = 10,544.1). However, the BIC indicated the superiority of Model 3, thus Model 3 was chosen as interpretable model as it showed acceptable fit across the majority of indices.

The final model (Figure 1) indicates that general self-efficacy was negatively associated with AjD symptom severity while dysfunctional disclosure was positively associated with AjD symptom severity. The association between loneliness and AjD symptom severity was positive and marginally significant. Reappraisal was positively associated with general self-efficacy. Perceived social support was negatively associated with loneliness and dysfunctional disclosure. Negative support resources were positively associated with loneliness and dysfunctional disclosure. Based on the modification indices, we identified a negative association between loneliness and general self-efficacy and a significant correlation between the residual variances of loneliness and dysfunctional disclosure (*r =* .30, *p <* .001).

### 3.3. Logistic regression

Table 3 shows the results of the binary logistic regression analysis with AjD diagnostic status as outcome. It showed a significant fit with the data (χ^2^(10, *N* = 316) = 88.50, *p *< .001). Loneliness, dysfunctional disclosure, perceived social support, and negative support resources were significantly, and positively associated with a higher probability of an AjD diagnosis. General self-efficacy showed a significant, and negative association with the outcome. Age showed a marginally significant and positive association with the probability of AjD diagnosis. In total, 36% of the variance could be explained by the variables included in the model (*R^2^*_Nagelkerke_ = .36).

**Table 2.** Correlation between study variables (Pearson coefficient) (*N* = 321).

	Age	Self-efficacy	Suppression	Reappraisal	Loneliness	Disclosure	Perceived social support	Positive support resources	Negative support resources
AjD symptom severity	.15**	−.35***	.07	.00	.40***	.68***	−.22***	−.06	.30***
Age	-	−.01	−.03	.13*	−.06	.02	−.04	.00	−.08
Self-efficacy		-	−.17**	.25**	−.40***	−.24***	.42***	.14*	−.33***
Suppression			-	.14*	.17*	.11	−.31***	−.22***	.11
Reappraisal				-	−.02	.07	.24***	.19**	−.10
Loneliness					-	.39***	−.42***	−.23***	.37***
Dysfunctional disclosure						-	−.19**	−.05	.30***
Perceived support							-	.48***	−.33***
Positive support resources								-	−.12*

* *p* < .05; ** *p* < .01; *** *p* < .001

**Table 3.** Logistic regression results for the diagnosis of adjustment disorder (*n* = 316).

			95% CI	
	β	OR	Lower	Upper
Sex (male)	−.02	0.88	0.46	1.68
Age	.06*	1.03	1.00	1.06
Self-efficacy	−.10**	0.89	0.82	0.97
Reappraisal	−.01	0.97	0.74	1.27
Suppression	−.03	0.89	0.67	1.19
Loneliness	.10**	1.44	1.11	1.85
Dysfunctional disclosure	.16***	1.11	1.06	1.15
Perceived support	.13**	2.93	1.47	5.85
Positive support resources	−.01	0.90	0.51	1.60
Negative support resources	.07*	1.96	1.05	3.65

R^2^ = .36 (Nagelkerke) .25 (Cox & Snell). Model χ^2^ = 88.50, *p* < .001

* *p* < .05; ** *p* < .01; *** *p* < .001

## 4. Discussion

The current study aims to contribute to the still sparse research on adjustment disorder by applying the socio-interpersonal framework model to identify risk factors. The prevalence of AjD according to the ICD-11 definition at 25.6% found in this sample showed that involuntary job loss significantly affects the well-being of the individuals concerned, and that a significant proportion develop symptoms of a diagnosable disorder. As can be seen by the high co-occurrence of other stressors, such as financial troubles or family conflicts, job loss has a multitude of implications and it can be accompanied by a disruption of other important areas of life.

Based on the socio-interpersonal framework model, we identified loneliness and dysfunctional disclosure as being associated with AjD symptom severity. Both mediated the relationship between perceived social support and AjD symptomatology, and between negative support resources and AjD symptomatology. This supports the assumption of different layers and differential influences in the model. The conceptualization of feelings of loneliness and dysfunctional disclosure in the present study relate to the social reality of the patients while the social support variables reflect interactive phenomena (Maercker & Horn, 2013). Consequently, loneliness and dysfunctional disclosure would be stronger associated with psychopathological symptoms, such as preoccupation with the stressor and failure to adapt, than social support (Maercker & Horn, 2013). The significant association between general self-efficacy and loneliness in explaining AjD symptom severity is in line with the view that the socio-interpersonal framework model adds to previous research that mostly focused on intrapersonal processes (Maercker & Horn, 2013).

Against expectations and in contrast to previous findings (Maercker et al., 2016), perceived social support was positively associated with AjD diagnostic status, indicating a higher probability of AjD diagnosis with higher perceived social support. This could be explained by the fact that in high stress situations people activate their social resources to regulate emotion (Lakey & Orehek, 2011). It might be that those people suffering more under the job loss rely more on their social contacts and therefore perceive their social support as higher. Another explanation of this finding could be the presence of a suppressor effect as perceived social support showed no association with diagnostic status in the univariate analysis in preparation of the logistic regression. A suppression effect in this case could mean that due to another predictor, e.g. loneliness, the association between social support and AjD symptom severity gets stronger. As we used multiple measures of social support, one possible explanation of a suppression effect could be multicollinearity in the data. However, the tolerance and variance inflation factor indicated no sign of multicollinearity. Due to concerns regarding power in the logistic regression, we were not able to test interaction effects between the independent variables, which could shed further light into possible suppression effects. The likelihood of suppressor effects and the role of perceived social support in the development of AjD should therefore be subject to future research.

One noticeable finding of the present study was the strong link between dysfunctional disclosure and AjD. A strong association between dysfunctional disclosure and symptoms of maladjustment to stress has been reported in previous studies (Fankhauser et al., 2010; Krutolewitsch et al., 2016) and can be explained by both theoretical assumptions and by measurement issues. Early theories assume that disclosure of experiences reduces stress through restructuring and reorganizing contents of the experience (Pennebaker, 1989). In stress-response syndromes, recurrent distressing thoughts are assumed to occur when stressful information is represented in active memory but not completely integrated into an individual’s cognitive schema (Horowitz, 1986). In the ICD-11 AjD definition recurrent distressing thoughts are reflected in preoccupation with the stressor. Not disclosing experiences or disclosing them in a dysfunctional way might thus interfere with the integration of the stressful experience into the self-concept and lead to preoccupation with it. Furthermore, the DTQ measure includes a scale of emotional reactions while disclosing. This scale assesses reactions such as tension, sadness, trembling, and exhaustion during or after disclosure. These reactions are to a certain extent similar to symptoms that individuals experience when they encounter problems of an adjustment disorder. The correlation between both measures suggest that there is some similarity between adjustment disorder symptoms and dysfunctional disclosure, however they can still be considered separate constructs. Future research could focus on the relationship between disclosure and AjD symptom development by focusing on different aspects of the disclosure process.

One limitation of the present study is the cross-sectional nature of the data used for the analyses. All results are based on associations; hence all predictions in the two models were purely statistical. There may be also reverse effects of AjD on the processes that we investigated, and we cannot entirely disentangle cause and effect. Further analyses are planned for the longitudinal part of the study. Also, we were not able to collect pre-job-loss data. To separate cause and effect of the event, a prospective longitudinal design would be needed. In addition, the data were mainly recorded by self-report questionnaires. The respective information still represents the personal view of the individual, which in particular makes the differentiation between intrapersonal and interpersonal processes harder. Moreover, one should bear in mind that our selection of intra- and interpersonal variables is not exhaustive. Accounting for the interaction of an individual with its environment is still neglected in clinical psychology (Maercker & Horn, 2013) and the socio-interpersonal framework wants to stress these contextual factors in psychopathology. Future studies should consider more objective measures and incorporate a more diverse set of variables. Job loss in an industrial country with high employment rates is of course a phenomenon that is different from other conditions of unemployment around the world. The socio-interpersonal model should be considered in future research on stress-related disorders and be applied to different contexts of work-related or economic strains.

This paper transferred a model on etiological factors of stress-response syndromes to the AjD context. It should be taken into consideration that there are also quantitative and qualitative differences between those disorders regarding presenting symptoms and precipitating life events (Maercker et al., 2013). As AjD has often been a hybrid of depressive and anxiety symptoms before (Fei, Ospedaliero, & Careggi, 2014), the new conceptualization aims at AjD as a self-sufficient diagnosis. Consequently, research should also focus on differential predictive factors and on finding pathognomonic risk factors for AjD.

## 5. Conclusion

The transition to unemployment creates a significant burden to the majority of individuals affected. Several processes that are associated with worse mental health outcome after job loss could be identified in the present study. A broader awareness and a deeper understanding of impairments in the unemployed population could lead to better service provision. Our findings support basic assumptions of the socio-interpersonal framework model for stress-response syndromes, supporting the new conceptualization of adjustment disorder. The integration of contextual factors in the understanding of the disorder can deepen our understanding of reactions to stressful life events and lead to more effective interventions.
